# The Sun, N. Y., for 1877

**Published:** 1877-02

**Authors:** 


*The Sun, New York,* 1877.—The different editions of The Sun during the next year will be the same as during the year that has just passed. The daily edition will on week days be a sheet of four pages, and on Sundays a sheet of eight pages, or 56 broad columns; while the weekly edition will be a sheet of eight pages of the same dimensions and character that are already familiar to our friends.

The Sun will continue to be the strenuous advocate of reform and retrenchment, and of the substitution of statesmanship, wisdom and integrity for hollow pretence, imbecility, and fraud in the administration of public affairs. It will contend for the government of the people by the people and for the people, as opposed to government by frauds in the ballot-box and in the counting of votes, enforced by military violence. It will endeavor to supply its readers—a body now not far from a million of souls—with the most careful, complete, and trustworthy accounts of current events, and will employ for this purpose a numerous and carefully selected staff of reporters and correspondents. Its reports from Washington, especially, will be full, accurate, and fearless; and it will doubtless continue to deserve and enjoy the hatred of those who thrive by plundering the Treasury or by usurping what the law does not give them, while it will endeavor to permit the confidence of the public by defending the rights of the people against the encroachments of unjustified power.

The prices of the daily Sun will be 55 cents a month or $6.50 a year, post paid, or with the Sunday edition $7.70 a year.

The Sunday edition alone, eight pages, $1.20 a year, post paid.

The Weekly Sun, eight pages of 56 broad columns, will be furnished during 1877 at the rate of $1 a year post paid.

The benefit of this large reduction from the previous rate for the Weekly can be enjoyed by individual subscribers without the necessity of making up clubs. At the same time, if any of our friends choose to aid in extending our circulation, we shall be grateful to them, and every such person who sends us ten or more subscribers from one place will be entitled to one copy of the paper for himself without charge. At one dollar a year, postage paid, the expenses of paper and printing are barely repaid; and, considering the size of the sheet and the quality of its contents, we are confident the people will consider the Weekly Sun the cheapest newspaper published in the world, and we trust also one of the very best. Address, The Sun, New York City, N. Y

